# Alteration in Endometrial Proteins during Early- and Mid-Secretory Phases of the Cycle in Women with Unexplained Infertility

**DOI:** 10.1371/journal.pone.0111687

**Published:** 2014-11-18

**Authors:** Murli Manohar, Huma Khan, Vijay Kumar Sirohi, Vinita Das, Anjoo Agarwal, Amita Pandey, Waseem Ahmad Siddiqui, Anila Dwivedi

**Affiliations:** 1 Division of Endocrinology, CSIR-Central Drug Research Institute, Lucknow, Uttar Pradesh, India; 2 Department of Obstetrics & Gynaecology, King George’s Medical University, Lucknow, Uttar Pradesh, India; 3 Department of Biochemistry, Jamia Hamdard (Hamdard University), New Delhi, India; Konkuk University, Republic of Korea

## Abstract

**Background:**

Compromised receptivity of the endometrium is a major cause of unexplained infertility, implantation failure and subclinical pregnancy loss. In order to investigate the changes in endometrial protein profile as a cause of unexplained infertility, the current study was undertaken to analyze the differentially expressed proteins of endometrium from early-secretory (LH+2) to mid-secretory phase (LH+7), in women with unexplained infertility.

**Methods:**

2-D gel electrophoresis was performed to analyze the proteomic changes between early- (n = 8) and mid-secretory (n = 8) phase endometrium of women with unexplained infertility. The differentially expressed protein spots were identified by LC-MS analysis and validated by immunoblotting and immuno-histochemical analysis in early- (n = 4) and mid-secretory (n = 4) phase endometrium of infertile women. Validated proteins were also analyzed in early- (n = 4) and mid-secretory (n = 4) phase endometrium of fertile women.

**Results:**

Nine proteins were found to be differentially expressed between early- and mid- secretory phases of endometrium of infertile women. The expression of Ras-related protein Rap-1b, Protein disulfide isomerase A3, Apolipoprotein-A1 (Apo-A1), Cofilin-1 and RAN GTP-binding nuclear protein (Ran) were found to be significantly increased, whereas, Tubulin polymerization promoting protein family member 3, Superoxide dismutase [Cu-Zn], Sorcin, and Proteasome subunit alpha type-5 were significantly decreased in mid- secretory phase endometrium of infertile women as compared to early-secretory phase endometrium of infertile women. Validation of 4 proteins viz. Sorcin, Cofilin-1, Apo-A1 and Ran were performed in separate endometrial biopsy samples from infertile women. The up-regulated expression of Sorcin and down-regulated expression of Cofilin-1 and Apolipoprotein-A1, were observed in mid-secretory phase as compared to early-secretory phase in case of fertile women.

**Conclusions:**

De-regulation of the expression of Sorcin, Cofilin-1, Apo-A1 and Ran, during early- to mid-secretory phase may have physiological significance and it may be one of the causes for altered differentiation and/or maturation of endometrium, in women with unexplained infertility.

## Introduction

Human endometrium undergoes a series of morphological and molecular changes during the transition from proliferative to secretory phase, under the influence of ovarian steroids [1], [2]. After ovulation, endometrium is under the influence of progesterone which undergoes differentiation and other morphological changes e.g. appearance of vacuoles, and formation of spiral arteries take place to develop the secretory endometrium [3]. During secretory phase, endometrium becomes receptive for a short period of time (spanning between days 20 and 24 of the menstrual cycle) to allow the attachment of developing embryo [4]. Intricate network of signalling molecules (hormones, cell adhesion molecules, cytokines and growth factors etc.) is known to be involved in embryo-uterine interaction and plays critical role in embryo attachment [1], [2]. Any alteration in cascade of these signalling mechanisms caused pregnancy loss, implantation failure or infertility [5].

Unexplained infertility accounts for approximately 25–30% of all known female infertility which is represented in a subset of infertile women having normal ovulatory cycles, normal hormonal profile and no evident reasons for infertility [6]. Compromised receptivity of the endometrium is a major cause of unexplained infertility, implantation failure and subclinical pregnancy loss. In women, unexplained infertility has been coupled with a range of cellular and molecular defects in the endometrium [7]–[9]. Several genomics and proteomics approach based studies have revealed large number of differentially regulated genes/proteins by comparing pre-receptive (LH+2) and receptive phase (LH+7) endometrium of fertile women [10]–[13]. Although, these studies have provided a large number of molecular candidates that are important during endometrial receptivity and some of them have been established as receptivity markers for fertile endometrium but to date the causes of unexplained infertility are not well explored. A few genomics and proteomics approach-based studies have explored the molecular targets responsible for the cause of recurrent implantation failure and unexplained infertility [14]–[16]. Although, the outcome from these studies have broadened our knowledge to understand the molecular mechanism involved in unexplained infertility, actual causes of unexplained infertility remain largely unknown.

With a view to explore the molecular basis of unexplained infertility, the present study was undertaken to generate 2D map of endometrial proteins during secretory phase of women with unexplained infertility and to analyze whether proteomic profile of endometrium differs from early- (LH+2) to mid-secretory (LH+7) phase. To our knowledge this is the first study to report the differentially expressed endometrial proteins between early-secretory (LH+2) and mid-secretory (LH+7) phase in women with unexplained infertility. Results of this study might be helpful in understanding the molecular mechanism involved in unexplained infertility and defective endometrial receptivity.

## Materials and Methods

### Ethics Statement

Human endometrial biopsy samples were obtained for research after written consent from each patient and the study was approved by the Institutional Ethics Committee of King George’s Medical University, Lucknow, India.

### Sample collection and patient details

Endometrial biopsies were taken by using pipelle catheters (Genetics, Belgium) under sterile condition and immediately frozen at −80°C until used. For proteomic study, 16 human endometrial biopsy samples were collected from different 16 women once during early-secretory (LH+2) (n = 8) and mid-secretory (LH+7) (n = 8) phases from infertile women with unexplained infertility. The detection of LH in morning urine (Donacheck ovulacion; Novalab Iberica, S.A.L., Coslada, Madrid, Spain) was used to determine the day of the LH surge (day LH+0). Additional eight endometrial samples (4 from each group, collected once) were collected from different infertile women outside the sample cohort, for the validation by western blot and immunohistochemistry, to confirm the biological significance of the findings. Another eight endometrial biopsy samples were collected once from different fertile women, during early-secretory (LH+2) (n = 4) and mid secretory (LH+7) (n = 4) phases of menstrual cycle. Histological dating of each sample was performed [17]. Serum progesterone levels as determined by immunoenzyme assay [18] were found to be 8.52±1.25 ng/ml for LH+2 stage and 18.16±1.32 ng/ml for LH+7 stage in infertile patients. A specific written consent was obtained from each patient, and the study was approved by the ethics committee of King George’s Medical University, Lucknow.

To determine the clinical state of infertile women, the complete examination and investigation of all infertile couples were carried out (Table S1). All women were aged 25–35 years and their cycle lengths were within normal range (27–29 days). The female patients had normal functioning fallopian tubes which were confirmed by HSG/SSG and if required diagnostic laparoscopy and hysteroscopy was done. Normal ovulatory function and absence of bacterial vaginosis has been confirmed. The test for systemic diseases e.g. diabetes was found to be negative, endometrial biopsies were negative for tuberculosis. The male partner had a normal sperm count. The couples have been trying to conceive for at least one year. Thus, the infertility investigation had not revealed any cause of the infertility.

### Exclusion criteria

The women having uterine abnormalities like leiomyomas, polycystic ovarian syndrome, endometriosis, hydrosalpinx, acute infection PID, vaginitis, male factor infertility were excluded from the study. Those who had received steroid hormone therapy in the last six months were also excluded from the study (Table S2).

The fertile women all had proven parity and were presenting for tubal ligation or assessment for reversal of tubal ligation.

Biopsy samples from only women with normal cycle and hormonal profile were collected. All patient groups were age matched and cycling (Table S3).

### Sample preparation for two dimensional polyacrylamide gel electrophoresis

10% homogenate was prepared in sample lysis buffer (7 M urea, 2 M thiourea, 4% CHAPS (3- (3-Cholamidopropyl) dimethylammonio)-1-propanesulfonic acid) and 20 mM Tris-pH 8.5), 1 mM EDTA, containing 50 mM DTT) in ice cold condition [19]–[22]. The whole tissue homogenate contained luminal epithelium, glandular epithelium, stroma, and vessels. Tissue homogenate was centrifuged and protein estimation was done [23].

### Isoelectric focusing

IPG strips (11 cm) were rehydrated with proteins (200 µg), mixed in rehydration buffer (8 M urea, 2% w/v CHAPS, 130 mM DTT, 0.002% bromophenol blue) and IPG buffer (11 µl) in a total volume of 210 µl before subjecting to IEF [23], [24]. The rehydrated strips (pH 3–10) were focused at 50 µA per strip in a multiphor-II electrophoresis unit (GE Healthcare, BUCKS, UK) up to 14000 V h at 20°C (500 V for 30 min linear gradient, 1000 V for 10 min, 8000 V for 3 h, 8000 V for 6 h and 8000 V for 7 h).

### SDS-PAGE

Following focusing, the strips were incubated in equilibration buffer (50 mM Tris/HCl, pH 8.8, 6 M urea, 30% (v/v) glycerol, 2% (w/v) SDS, 0.01% (w/v) bromophenol blue) for 45 min placed over the resolving gels, covered with sealing gel (0.5% agarose in SDS electrophoresis buffer) and SDS-PAGE was carried out on 12.5% polyacrylamide gels over night in a Bio-Rad Dodeca cell (Richmond CA). Proteins were visualized by silver staining as reported previously [24], [25]. In brief, gels were fixed in methanol: acetic acid: water in the ratio of 50: 5: 45, for 2 h. Gels were rinsed with double distilled water (DDW) twice and kept in DDW for 1 h. Gels were then incubated with sensitizing solution (0.02% sodium thiosulphate) for 1–2 min with constant shaking. Further, the gels were incubated in 0.1% AgNO_3_ solution in dark for 30 min and spots were developed by a solution of 2% Na_2_CO_3_ and 35% HCHO for 5 min. 1% Acetic acid was used immediately to avoid the over staining of gels [24], [25]. Coommassie brilliant blue (R-250) staining was also performed to confirm the differentially expressed protein spots which were observed in silver stained gel. Equal amount of protein was loaded in each experiment. Similar procedure for analysis/staining was used to minimize the variations arising due to ‘sample loading’, ‘staining procedures’ and ‘run-to-run’ variability.

### Image capture and analysis

The silver stained 2D gels of early-secretory (LH+2) (n = 8) and mid-secretory phase (LH+7) (n = 8) endometrium of infertile women were scanned and analyzed by using image master-2D-Platinum version 7. With this view, a total of eight independent experiments were performed. To allow quantitative comparison between 2D gels, all the gel images were aligned properly, calibrated and normalized using Image Master 2D platinum software (Amersham Biosciences). Total 16 gels were analyzed (8 from each group) and volume of each spot was normalized against the sum total of volume of all detectable spots in the 2D gel, this normalization was performed by Image Master 2D platinum software that corrects for any minor differences in protein loading among replicate gels. The background in silver-stained gels has been subtracted at the time of gel analysis with the help of Image Master 2D platinum software to avoid any possible error over protein spot intensity. The volume of all the spots from independent sets of experiments were taken (Table S4). To calculate fold change, the spot volume of early-secretory phase endometrium was divided with spot volume of mid-secretory phase endometrium of infertile women. Only protein spots that changed ≥1.5-fold and were consistently altered in the same manner in all eight experiments, were considered to be differentially expressed. One-way ANOVA along with unpaired ‘t’ test was used to determine the significance of data between the two groups.

### Protein identification by liquid chromatography-mass spectrometric analysis (LC-MS)

The gel pieces were digested with trypsin and cut into size of 1 mm, washed with 500 µl of H_2_O followed by 500 µl of 25 mM ammonium bicarbonate in 50% acetonitrile for 60 min. The gel pieces were dehydrated by the adding 500 µl of acetonitrile. Disulfide bonds were cleaved by incubating the samples for 60 min at 56°C with 200 µl of 10 mM DTT in 25 mM ammonium bicarbonate buffer. Alkylation of cysteines was performed by the addition of 200 µl of 55 mM iodoacetamide in 25 mM ammonium bicarbonate buffer and incubation of the samples for 45 min at room temperature in darkness. Gel bands were washed with 25 mM ammonium bicarbonate buffer and dehydrated with 500 µl of acetonitrile. Gel pieces were covered with trypsin solution (10 ng/µl in 25 mM ammonium bicarbonate buffer). After 30-min incubation on ice, the remaining trypsin solution was removed, and 25 µl of 25 mM ammonium bicarbonate was added. Proteolysis was performed overnight at 37°C and stopped by adjusting the samples to 5% formic acid. Further, these peptides were analyzed by electrospray ionization mass spectrometry using the Ultimate 3000 nano HPLC system (Dionex) coupled to a 4000 Q TRAP mass spectrometer (Applied Biosystems). Tryptic peptides were loaded on to a C18 PepMap100, 3 µM (LC Packings) and separated with a linear gradient of water/acetonitrile/0.1% formic acid (v/v) at flow rate of 0.3 µl/min and gradient elution time was 45 minutes. Data were analyzed by using Mascot sequence matching software (Matrix Science) with Ludwig NR database and taxonomy set to human [26]–[29]. Changes were deemed significant at a false discovery rate (FDR) corrected p-value of <0.05 using a standard Benjamini-Hochberg correction [30]–[33]. Details of LC-MS data have been given in Table S5, Figures S1 to S9. Standard search parameters were: type of search, MS/MS ion search; enzyme, trypsin; external calibration,100 ppm; variable modifications, oxidation (M); mass values, monoisotopic; protein mass, unrestricted; peptide mass tolerance, ±1.2 Da; fragment mass tolerance, ±0.6 Da, and up to 1 missed cleavage was allowed.

### Western Blot Analysis

The whole tissue lysate of human endometrial samples from pre-receptive (LH+2) (n = 4), receptive phase (LH+7) (n = 4) endometrium of infertile women with unexplained infertility and pre-receptive (LH+2) (n = 4), receptive phase (LH+7) (n = 4) endometrium of fertile women were prepared by homogenizing in RIPA buffer described by Awasthi et al (2007) [34]. 30 µg of protein/lane were run on 12% sodium dodecyl sulfate-polyacrylamide gel electrophoresis. Proteins were transferred on polyvinylidene difluoride membrane and blocked with 5% skimmed milk. Membranes were incubated overnight with primary antibody anti-Sorcin (39 M, sc-100859, Santacruz), anti-Ran (A7, sc-271376, Santacruz), anti-Apo-A1 (B-10, sc-376818, Santacruz) anti-Cofilin-1 (H-12, sc-32158, Santacruz) and anti-β-actin (N-21, sc-130656, Santacruz) of human origin and followed by incubation with secondary horseradish peroxidase conjugated antibodies. Bands were detected using enhanced chemiluminescence detection system (Amersham Biosciences). Quantitation of band intensity was performed by densitometry using Quantity One software (v. 4.5.1) and a Gel Doc imaging system (Bio-Rad).

### Immunohistochemistry

Immunohistochemical analysis was performed with minor modifications as described previously [34]. In brief, formalin-fixed and paraffin-embedded endometrial biopsies sections were de-paraffinized and rehydrated. The sections were treated with 0.5% H_2_O_2_ in deionized water for 5 min to block endogenous peroxidase activity and were blocked with 5% bovine serum albumin along with normal goat serum for 2 h. followed by primary antibodies having reactivity in human 1∶500, anti-Sorcin (39 M, sc-100859, Santacruz), anti-Ran (A7, sc-271376, Santacruz), anti-Apo-A1 (B-10, sc-376818, Santacruz) and anti-Cofilin-1 (H-12, sc-32158, Santacruz) for 48 h at 4°C temperature. For the negative control, mouse/goat IgG (Santacruz) was used in place of primary antibody. These sections were incubated with biotin labelled anti-mouse/anti-goat secondary antibodies (1∶1000) (Sigma Aldrich) for 2 h at room temperature, followed by Streptavidin (1∶1000) (Invitrogen) incubation for 1 h. Staining was achieved with 3, 3- diaminobenzidine (Sigma-Aldrich) for 5 min and counterstained by hematoxylin for 1 min after washing with double distilled water, slides were mounted with DPX (Sigma-Aldrich). The staining intensity of all these proteins in glandular epithelium, luminal epithelium and stromal compartment were quantified by image analysis software Image-Pro Plus 4.0 (Maryland, USA) and results were expressed as % image analysis score [34].

### Statistical analysis

One-way ANOVA along with unpaired ‘t’ test was used to determine the significance of data between the two groups. The data are expressed as means ± SE, p value less than 0.05 was considered as significant.

## Results

### Proteomic profile of early-secretory (LH+2) and mid-secretory phase (LH+7) endometrium of women with unexplained infertility

**Figure 1:**
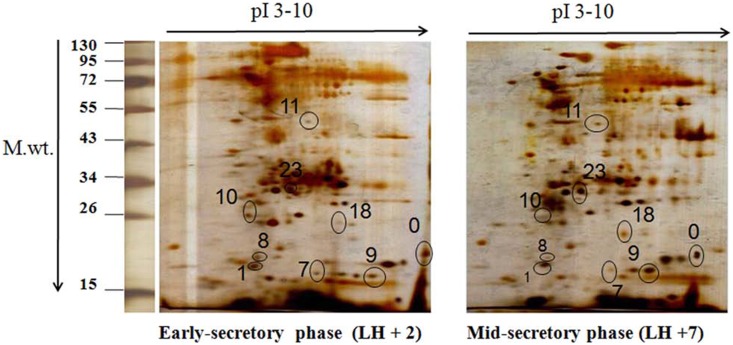
2D-PAGE of early-secretory (LH+2) and mid-secretory (LH+7) phase endometrium of women with unexplained infertility. Representative gel images are shown here. The first dimension was performed by IEF on IPG strips over a range of pI 3–10, the second dimension on 12.5% SDS-PAGE gels and the proteins were visualized by silver staining. 9 differentially altered protein spots were identified by image master 2D platinum software and number denotes spot ID (0–23). LH+2 = 2 days after luteinizing hormone surge, LH+7 = 7 days after luteinizing hormone surge.

Comparison of proteomic profile of early-secretory (LH+2) and mid-secretory phase (LH+7) endometrium of women with unexplained infertility were performed by 2D-PAGE analysis. Whole proteins of human endometrial tissue from early-secretory and mid-secretory phase, following 2D-PAGE, were resolved into a number of proteins in the molecular weight range of 15–130 kDa and pI of 3–10 (Figure 1). A total of 78 protein spots were observed and among them 18 protein spots were aligned in all gels. The proteins displaying significant (p<0.05) expression changes across all the gel images were considered to have altered expression levels having ≥1.5 fold change in their expression. Only 9 proteins showed significant abundance changes (FDR corrected p-value <0.05 [30]) between early- and mid- secretory endometrium (5 increased and 4 decreased in the mid- versus early-secretory phase) by using Image master-2D- platinum software version 7 (Figure 1).

**Figure 2:**
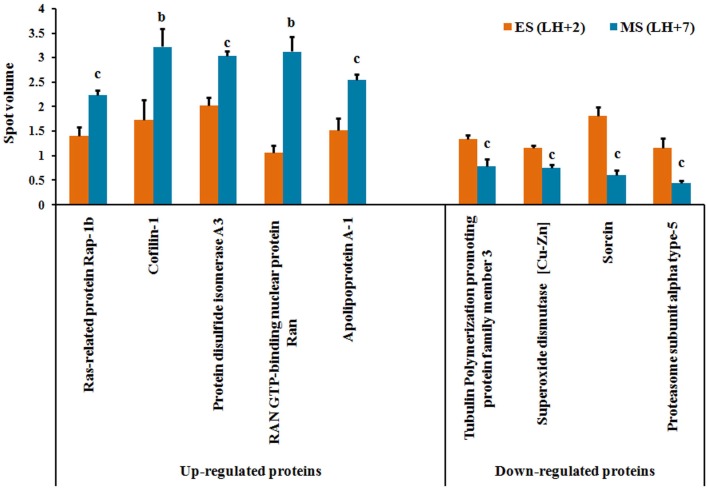
Various differentially expressed proteins. Bar diagram showing 9 differentially expressed proteins between early-secretory (LH+2) and mid-secretory (LH+7) phase endometrium from women with unexplained infertility. Values were expressed as mean ± SE, n = 8. P values are (b) p<0.01 and (c) p<0.05 versus early-secretory phase (LH+2). ES = early-secretory phase, MS = mid-secretory phase, LH+2 = 2 days after luteinizing hormone surge, LH+7 = 7 days after luteinizing hormone surge.

These 9 consistently appearing protein spots were excised from the silver stained 2D gels and processed for identification by LC-MS analysis. Details of these differentially expressed proteins have been shown in Table 1 and Figure 2. The 5 increased proteins were identified as Ras-related protein Rap-1b, Protein disulfide isomerase A3, Apolipoprotein-A1 (Apo-A1), Cofilin-1 and RAN GTP-binding nuclear protein (Ran). Apart from this, the 4 decreased proteins were identified as Tubulin polymerization promoting protein family member-3, Superoxide dismutase [Cu-Zn], Sorcin, and Proteasome subunit alpha type-5.

**Table 1:** Details of differentially expressed proteins identified by LC-MS analysis and comparison thereof with previous reports.

S. No.	SampleID	ProteinID	Protein identifiedby LC- MSanalysis	Proteinscore	Matchedpeptide	Coverage	MW(KDa)/PI	Expressionand foldchange	Biologicalfunction	Reports frompreviousstudies
1	0	IPI00306413	Tubulin-polymerizationpromoting proteinfamily member 3	1086	488	0.579	19.14/10.07	Down(1.65 fold)	Cytoskeletalprotein	-
2	1	IPI00015148	Ras-relatedproteinRap-1b	713	426	0.152	21.03/5.39	Up(1.58 fold)	GTPaseactivity	Up-regulatedinendometriosis(Matsuzaki et al., 2005)
3	7	IPI00218733	Superoxidedismutase[Cu-Zn]	717	224	0.558	16.15/6.07	Down(1.52 fold)	Antioxidantsystem	Up-regulatedinmid-secretoryphase offertilewomen(Sugino etal., 1996);Up-regulatedin LH+7phase of fertile women (Riesewijk et al., 2003); Up-regulated in LH+7 phase of fertile women (Li et al., 2011)
4	8	IPI00027175	Sorcin	229	223	0.186	21.94/5.21	Down(2.97 fold)	Ca^+2^signaling	Up-regulated in secretory phase of fertile women (Chen et al., 2009)
5	9	IPI00012011	Cofilin-1	642	208	0.355	18.71/8.29	Up(1.85 fold)	Cytoskeletalprotein	Down-regulated in secretory phase of fertile women (DeSouza et al., 2005); Decreased secretion in uterine fluid of mid-secretory phase (Scotchi et al., 2009)
6	10	IPI00291922	Proteasomesubunitalpha type-5	119	60	0.241	26.56/4.45	Down(2.59 fold)	Proteindegradation	Up-regulated in secretory phase of fertile women (DeSouza et al., 2005)
7	11	IPI00025252	ProteindisulphideisomeraseA3	1148	1354	0.316	57.14/6.28	Up(1.50 fold)	Molecularchaperone	Down-regulated in secretory phase of fertile women (Rai et al., 2009)
8	18	IPI00643041	RAN GTP-binding nuclearprotein (Ran)	512	302	0.310	24.57/7.59	Up(2.94 fold)	Nucleo-cytoplasmictransport	Reported in proliferative and secretory phase endometrium in fertile women; (Borthwick et al, 2003)
9	23	IPI00021841	Apolipoprotein A1	2947	410	0.835	30.75/5.5	Up(1.67 fold)	Lipidmetabolism	Up-regulation in RIF patient during mid-secretory phase endometrium (Brosens et al., 2010); Decreased secretion in uterine fluid of mid-secretory endometrium of infertile women (Hannan et al., 2010); Decreased secretion in uterine fluid of mid-secretory endometrium of fertile women (Scotchi et al., 2009)

Alteration in endometrial proteins during early- and mid-secretory phases of the cycle in women with unexplained infertility.

### Western blot analysis of Sorcin, Cofilin-1, Apolipoprotein-A1 and Ran GTP-binding nuclear protein

**Figure 3:**
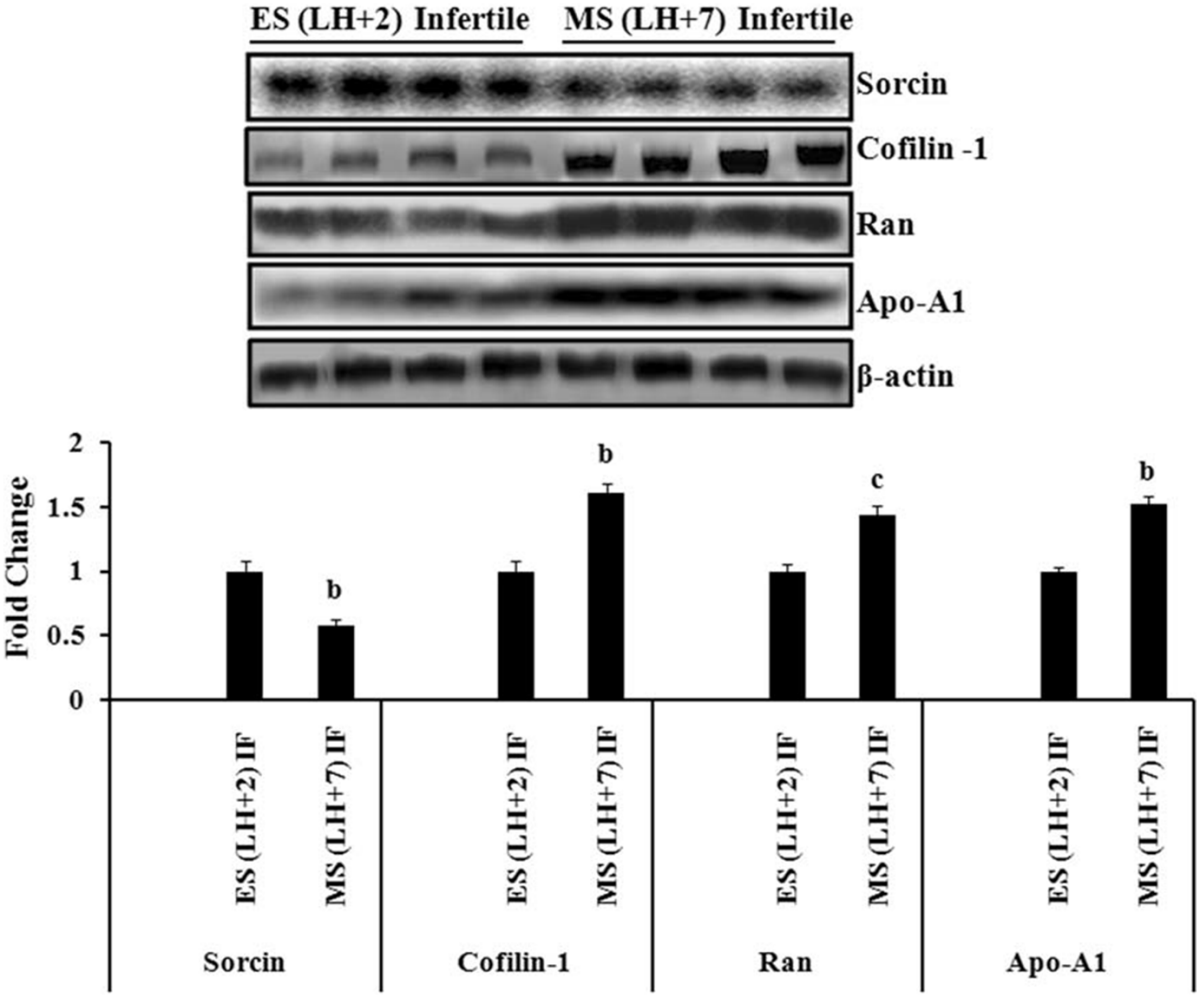
Validation of Sorcin, Cofilin-1, Apolipoprotein-A1 (Apo-A1) and Ran GTP-binding nuclear protein (Ran) in infertile women. Expression of Sorcin, Cofilin-1, Apo-A1 and Ran were analyzed by western blot analysis in early-secretory (LH+2) and mid-secretory phase (LH+7) endometrium of infertile women, which had not been previously analyzed in the 2D-PAGE analysis. The results matched the findings of 2D-PAGE analysis. Representative images (upper panel) of immunoblot of Sorcin, Cofilin-1, Apo-A1 and Ran have been shown. β-actin served as a loading control for normalization. Quantification of band intensity (lower panel) was performed by densitometric analysis by using Quantity One software (v. 4.5.1) and a Gel Doc imaging system (Bio-Rad). P values are (b) p<0.01 and (c) p<0.05 versus early-secretory phase (LH+2). Values are expressed as mean± SE; n = 4. ES = early-secretory phase, MS = mid-secretory phase, LH+2 = 2 days after luteinizing hormone surge, LH+7 = 7 days after luteinizing hormone surge.

To validate protein differences attained by 2D-PAGE, western blot analysis was performed for Sorcin, Cofilin-1, Apo-A1 and Ran GTP-binding nuclear protein (Ran) in another biopsy samples of early-secretory (n = 4) and mid-secretory phase (n = 4) endometrium from women with unexplained infertility. β-actin was used as a loading control to normalize protein abundance in all the experiments. Expression of Sorcin was found significantly (p<0.01) down-regulated in mid-secretory phase (LH+7) as compared to early-secretory phase endometrium. The expression of Cofilin-1, Apo-A1 and Ran were significantly (p<0.01, p<0.05) up-regulated in mid-secretory phase (LH+7) endometrium as compared to early-secretory phase (LH+2) endometrium from the women with unexplained infertility (Figure 3). The expression pattern of these proteins was found to be similar as observed in 2D-PAGE analysis.

**Figure 4:**
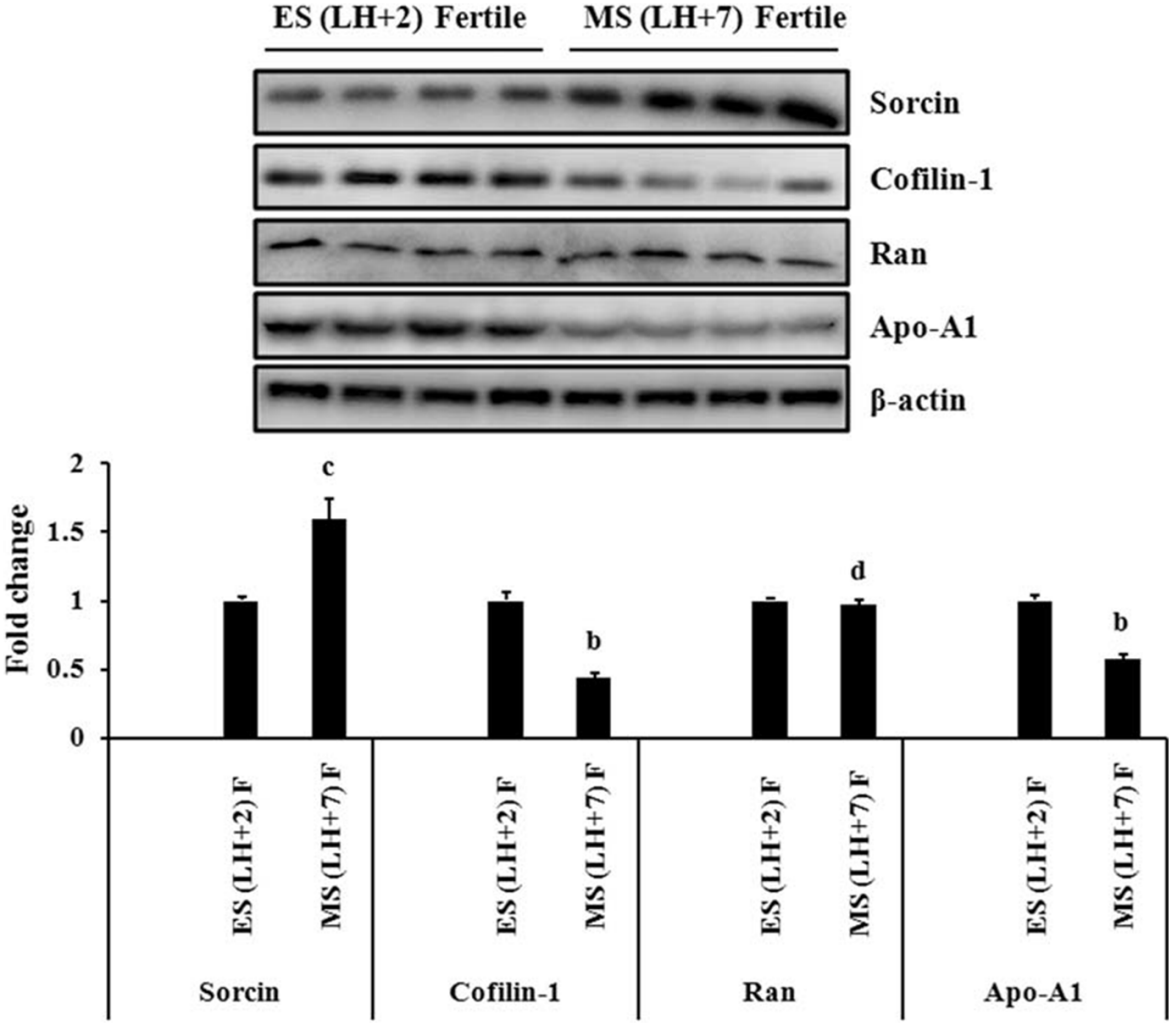
Immunoblot analysis of Sorcin, Cofilin-1, Apolipoprotein-A1 (Apo-A1) and Ran GTP-binding nuclear protein (Ran) in fertile women. Expression of Sorcin, Cofilin-1, Apo-A1 and Ran were checked in early-secretory (LH+2) and mid-secretory (LH+7) phase endometrium of fertile women. Representative images (upper panel) of immunoblot of Sorcin, Cofilin-1, Apo-A1 and Ran have been shown. β-actin served as a loading control for normalization. Quantification of band intensity (lower panel) was performed by densitometric analysis by using Quantity One software (v. 4.5.1) and a Gel Doc imaging system (Bio-Rad). p-values are (b) p<0.01, (c) p<0.05 and (d) p>0.05 versus early-secretory phase (LH+2). Values are expressed as mean± SE; n = 4, ES = early-secretory phase, MS = mid-secretory phase, LH+2 = 2 days after luteinizing hormone surge, LH+7 = 7 days after luteinizing hormone surge.

In the endometrium of fertile women, the expression of Sorcin was significantly up-regulated in mid-secretory phase as compared to early-secretory phase whereas, the expression of Cofilin-1 and Apo-A1 was significantly down-regulated in mid-secretory phase. However, the expression of Ran was unaltered in both the phases of endometrium in fertile women (Figure 4).

### Immunohistochemistry of Sorcin, Cofilin-1, Apolipoprotein-A1 and Ran GTP-binding nuclear protein

**Figure 5:**
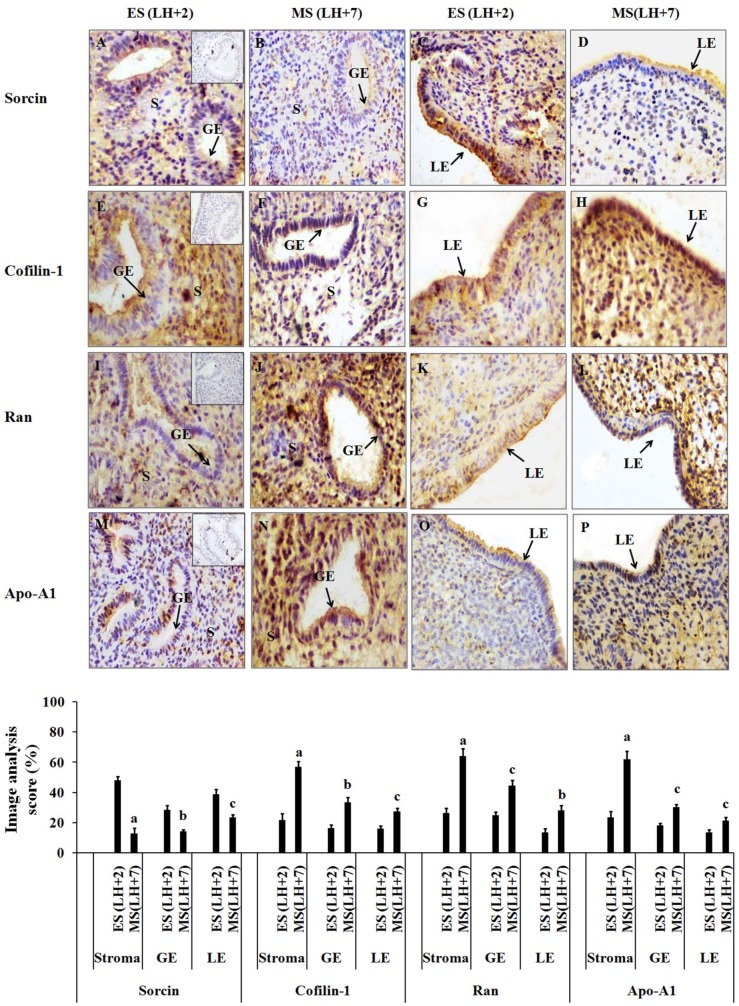
Immunohistochemical localization of Sorcin, Cofilin-1, Apolipoprotein-A1 (Apo-A1) and Ran GTP-binding nuclear protein (Ran) in infertile women. Representative images (upper panel) showing immunohistochemical localization of of Sorcin, Cofilin-1, Apo-A1 and Ran in early-secretory (LH+2) and mid-secretory (LH+7) phase endometrium from women with unexplained infertility. Lower panel shows image analysis of Sorcin, Cofilin-1, Apo-A1 and Ran in early-secretory and mid-secretory phase endometrium of infertile women. Staining intensity of all these proteins were quantified by image analysis software Image-Pro Plus 4.0 (Maryland, USA). Anti-mouse/anti-goat IgG was used as negative control and shown in inset (Fig. 4 A, E, I, M). Magnification X 400, bar = 25 µm, p-values are (a) p<0.001, (b) p<0.01 and (c) p<0.05 versus early-secretory phase (LH+2). Values are expressed as mean ± SE. n = 4 in all the groups. S = stroma, GE = Glandular epithelium, LE = Luminal epithelium.

To validate the observed protein abundance changes between early-secretory (LH+2) and mid-secretory phase (LH+7) endometrium from infertile women, immunohistochemistry of Sorcin, Cofilin-1, Ran GTP-binding nuclear protein (Ran) and Apo-A1 were carried out on individual endometrial biopsies following fixing in formalin. Image analysis revealed the down-regulated expression of sorcin in stroma (3.33 fold), luminal epithelium (LE) (1.6 fold) and glandular epithelium (GE) (2 fold) of mid-secretory phase (LH+7) as compared to stroma, LE, and GE of early-secretory phase (LH+2) endometrium of infertile women (Figure 5A–D). In case of Cofilin-1, up-regulated expression was observed in stroma (2.4 fold), LE (1.9 fold) and GE (2 fold) of mid-secretory phase (LH+7) as compared to early-secretory phase (LH+2) endometrium of infertile women (Figure 5E–H). Expression of Ran was up-regulated 2.32 fold in stromal cells, 1.86 fold in LE and 1.66 fold in GE of mid-secretory phase as compared to early-secretory phase endometrium of infertile women (Figure 5I–L). The expression of Apo-A1 was up-regulated in stromal cells (2.6 fold), LE (1.66 fold), and GE (1.57 fold) of mid-secretory phase as compared to early-secretory phase endometrium of infertile women (Figure 5M–P). The magnitude of difference in the expression of sorcin, cofilin-1, Ran and Apo-A1were higher in stroma than in LE and GE. No staining was observed in negative controls where primary antibody was substituted by control IgG (see inset in Figure 5A, E, I and M).

## Discussion

We have for the first time compared the protein profile between early-secretory (LH+2) and mid-secretory phase (LH+7) endometrium of women with unexplained infertility (UI) by using 2D-PAGE followed by LC-MS analysis. Nine consistent differentially expressed proteins were identified in mid- secretory phase (LH+7) endometrium as compared to early-secretory phase (LH+2) endometrium of women with unexplained infertility (Figure 1, Table 1). We have also compared our findings with previously published reports on cyclical changes in various endometrial proteins from ‘pre-receptive to receptive’ phase as well as from ‘proliferative to secretory’ phase in normal fertile women (Table 1).

Ras-related protein Rap-1b is a small GTPase and member of Ras superfamily. Rap-1b serve as a molecular switch and regulate several cellular processes, such as cell growth, cell cycle, angiogenesis, and differentiation [35], [36]. The increased expression of Rap-1b has been found in women with endometriosis as compared to early secretory phase endometrium from fertile women and, is regulated via RAS/RAF/MAPK and PI3K pathways [37]. In our study, endometrial expression of Rap-1b was increased during mid-secretory phase (LH+7) when compared to early-secretory phase (LH+2) in women with unexplained infertility. The increased expression of Rap-1b in mid-secretory phase of infertile women may be because of altered- or dysregulated- progesterone signalling and might also involve RAS/RAF/MAPK and PI3K pathways. However, the regulation of Rap-1b protein in relation to progesterone signalling is a matter of further investigation.

RAN GTP-binding nuclear protein (Ran), is also a member of Ras superfamily with GTPase activity and known to involved in nucleo-cytoplasmic transport of various protein and RNA across the nuclear pore [38], [39]. Higher level of Ran has been reported during progression and development of ovarian cancer cells [40]. In a genomic profile based study, presence of Ran has been found in proliferative as well as secretory phase endometrium of fertile women [11]. However, cyclic changes in Ran expression and hormonal regulation thereof is not explores as yet. In our study, the endometrial expression of Ran was found to be increased in mid-secretory phase as compared to that in early- secretory phase in unexplained infertility and the highest magnitude of change was observed in stromal cells. However, the expression of Ran was unaltered in fertile women in early- and mid-secretory phase endometrium. The increased expression of Ran during mid-secretory phase of infertile women might have a role in altering the progression and development of stromal cells of the endometrium.

Protein disulfide-isomerase A3 (PDIA3) is involved in the proper folding of proteins [41], [42]. The regulation of PDIA3 via IL-11/STAT3 signaling in rat uterine stromal cells has been found [43]. Whereas, the down-regulation of PDIA3 by siRNA resulted in decreased activation of STAT3 which is involved in cell proliferation and growth [44]. In our study, the expression of PDIA3 was increased in mid-secretory phase as compared to early-secretory phase endometrium of women with unexplained infertility. In a report, the decreased expression of PDIA3 was found in the endometrium of fertile women during secretory phase as compared to proliferative phase [19]. However in our study, the increased expression of PDIA3 during receptive phase/mid-secretory phase endometrium of infertile women might induce IL-11/STAT3 signalling pathway, which in turn cause proliferation and growth of endometrial cells. Nevertheless, more functional studies are required to support our hypothesis.

Apolipoprotein-A1 has anti-inflammatory property, and has ability to inhibit the synthesis of inflammatory mediators and cell adhesion molecules that might play crucial role at the time of implantation [15], [45], [46]. Previous report revealed decreased secretion of Apo-A1 in the uterine fluid of mid-secretory endometrium as compared to early-secretory endometrium of fertile women [47]. In the current study, the expression of Apo-A1 was found to be increased in mid-secretory phase as compared to early-secretory phase endometrium in infertile women. Significantly increased immunoreactivity of Apo-A1 was found in stromal compartment, followed by LE and GE, in mid-secretory phase endometrium of infertile women. However, the expression of Apo-A1 in mid-secretory phase of fertile women was down-regulated over the early-secretory phase endometrium in case of fertile women. This indicates that the down-regulated expression of Apo-A1 might be responsible for decidualization of stromal cells in the normal fertile women. Furthermore, Brosens *et al*, (2010) have revealed higher expression of Apo-A1 in the mid-secretary phase endometrium which might be a cause for recurrent implantation failure [15]. In contrary, the decreased expression of Apo-A1 was found in uterine lavage of women with unexplained infertility [16]. This difference in the expression pattern of Apo-A1 may be because of variation in experimental sample i.e. uterine lavage and the endometrial tissue. Another study carried out on genomic profiling at the time of embryo implantation in women with unexplained infertility, has also revealed the differential (up-regulated as well as down-regulated) expression of genes involved in lipid metabolism [14]. We hypothesize that up-regulated expression of Apo-A1 during receptive/mid-secretory phase of endometrium might have altered the normal level of inflammatory as well as cell adhesion molecules which may be responsible for inadequate progression of stromal cells to decidual cells thus causing infertility.

Cofilin-1, a critical modulator of the actin cystoskeleton, is associated with cell adhesion, cell invasion and angiogenesis [48]. Dysregulation of actin reorganization is suggested to be one of the causative factors in endometriosis [49]. Earlier proteomic -based study by DeSouza *et al*, (2005) have revealed the down-regulated expression of Cofilin-1 in fertile women during secretory phase as compared to proliferative phase [50]. Decreased secretion of Cofilin-1 was reported in uterine fluid of mid-secretory endometrium as compared to early-secretory endometrium of fertile women [47]. In our study, the endometrial expression of Cofilin-1 was up-regulated in mid-secretory phase as compared to early-secretory phase in case of unexplained infertility. Immunolocalization experiments revealed the up-regulated expression of Cofilin-1 in stroma, GE and LE in mid-secretory phase as compared to early- secretory phase endometrium of women with unexplained infertility. Interestingly, the expression of Cofilin-1 was observed to be down-regulated in mid-secretory phase as compared to early-secretory phase endometrium in case of fertile women. The low expression of Cofilin-1 during mid-secretory phase might be necessary for proper endometrial responsiveness to progesterone. During the secretory phase of fertile women, expression of several cytoskeletal proteins becomes altered [19]. The increased expression of Cofilin-1 in receptive phase endometrium of infertile women might be because of improper actin polymerization and depolymerization [48]. Apart from this, another cytoskeletal protein, Tubulin polymerization promoting protein family member 3 (TPPP3) was also identified with decreased expression in mid-secretory phase endometrium than in early-secretory phase in these infertile women. The detailed investigation of role of tubulins in development and differentiation of secretory phase of the endometrium is further warranted.

Sorcin, a calcium-binding protein modulates ryanodine receptor function and intracellular calcium release [51]. Earlier proteomic approach- based study revealed the up-regulated expression of sorcin in secretory phase as compared to mid-proliferative phase in endometrium of fertile women [20]. In our study, the expression of Sorcin was down-regulated in mid-secretory phase as compared to early-secretory phase as observed in women with unexplained infertility. Immunolocalization experiment showed the down-regulated expression of Sorcin in stroma in mid-secretory phase as compared to early-secretory phase in case of infertile women. In contrast, the expression of Sorcin was up-regulated in the mid-secretory phase as compared to early-secretory phase in fertile women. The altered regulation of Sorcin might cause imbalance in Ca^+2^ levels [52] and might result in inadequate functioning of endometrium leading to infertility. Whereas in fertile samples the expression of Sorcin was found to be up-regulated suggesting its essential role in receptivity. Thus, Sorcin might be required in the development of secretory endometrium and might play crucial role during the embryo implantation. However, further studies are required to establish the functional role of Sorcin in embryo implantation.

Superoxides play important role in reproductive processes involving embryonic development and implantation [53], [54]. In our study, in women with unexplained infertility, the expression of superoxide dismutase [Cu-Zn] was decreased during mid-secretory phase as compared to early-secretory phase endometrium. The earlier study in fertile women, revealed the higher activity of superoxide dismutase [Cu-Zn] during mid-secretory phase [55]. In a genomic profiling- based study, the increased expression of mitochondrial superoxide dismutase-2, was observed in receptive phase (LH+7) endometrium as compared to pre-receptive phase (LH+2) endometrium of fertile women [10]. In a proteomic study, up-regulation of superoxide dismutase (Mn) was found in the receptive phase as compared to pre-receptive phase endometrium of fertile women [13]. Furthermore, the reduced fertility rate has been found in female mice lacking superoxide dismutase [Cu-Zn] [56], [57]. It appears that reduced level of superoxide dismutase [Cu-Zn] during mid-secretory phase endometrium may be responsible for dysfunctional antioxidant system in infertile women. This suggested the possibility of endometrial receptivity improvement via augmenting antioxidant defences.

Proteasome subunit alpha type-5 (PSMA5) is a subunit of proteinase complex and participates in protein degradation [58], [59]. Since efficient protein degradation is critical for cell cycle progression and apoptosis [60]. In fertile women, the phenomenon of apoptosis in endometrium is common during secretory phase [61]. There might be a possibility of involvement of proteasomal complex in degradation of several proteins involved at the time of apoptosis during secretory phase [62]. A 2.1 fold higher ratio of PSMA5 was found in endometrium at secretory phase over proliferative phase in case of fertile women [50]. These evidences suggest the possible role of PSMA5 in apoptosis during secretory phase. In our study, PSMA5 expression was decreased in mid-secretory phase as compared to early-secretory phase endometrium. It may be speculated that the reduced expression of PSMA5 in women with unexplained infertility, during mid-secretory phase of the cycle, slows down the process of apoptosis and might cause improper differentiation of endometrial cells. However, future in-depth studies are required to confirm the role of PSMA5 during apoptosis in human endometrium.

Thus, 2D-PAGE analysis followed by LC-MS analysis of the endometrial proteins in women with unexplained infertility explored the proteins that may have functional significance during transition from early-secretory (pre-receptive) (LH+2) to mid-secretory (receptive) phase (LH+7) of the menstrual cycle. However, further functional studies will be required to define those proteins important for explaining the molecular basis of infertility. To conclude, the analysis of endometrial proteins provided a general survey of changes in protein expression which represents the first step toward elucidating the definite molecular conditions during unexplained infertility. Differential expression of certain proteins during mid-secretory phase may be responsible for endometrial receptivity- related defects and/or alteration in the process of endometrial differentiation during pre-receptive to receptive phase transition. Taken together, the results of this study might be helpful in understanding and exploring the molecular mechanism involved in unexplained infertility. In addition, it opens avenues for search of role of these proteins as novel players in endometrial receptivity.

## Supporting Information

Figure S1 **Details of LC-MS analysis of Tubulin-polymerization promoting protein family member-3.** (A) unique spectrum number, (B) unique peptide number, (C) Score delta (D), Charge, (E) fragment ion mass/Da, (F), peptide mass delta/Da.
(TIF)

Figure S2 **Details of LC-MS analysis of Ras-related protein Rap-1b.** (A) unique spectrum number, (B) unique peptide number, (C) Score delta (D), Charge, (E) fragment ion mass/Da, (F), peptide mass delta/Da.
(TIF)

Figure S3 **Details of LC-MS analysis of Superoxide dismutase [Cu-Zn].** (A) unique spectrum number, (B) unique peptide number, (C) Score delta (D), Charge, (E) fragment ion mass/Da, (F), peptide mass delta/Da.
(TIF)

Figure S4 **Details of LC-MS analysis of Sorcin.** (A) unique spectrum number, (B) unique peptide number, (C) Score delta (D), Charge, (E) fragment ion mass/Da, (F), peptide mass delta/Da.
(TIF)

Figure S5 **Details of LC-MS analysis of Cofilin-1.** (A) unique spectrum number, (B) unique peptide number, (C) Score delta (D), Charge, (E) fragment ion mass/Da, (F), peptide mass delta/Da.
(TIF)

Figure S6 **Details of LC-MS analysis of Proteasome subunit alpha type-5.** (A) unique spectrum number, (B) unique peptide number, (C) Score delta (D), Charge, (E) fragment ion mass/Da, (F), peptide mass delta/Da.
(TIF)

Figure S7 **Details of LC-MS analysis of Protein disulfide isomerase A3.** (A) unique spectrum number, (B) unique peptide number, (C) Score delta (D), Charge, (E) fragment ion mass/Da, (F), peptide mass delta/Da.
(TIF)

Figure S8 **Details of** **LC-MS analysis of RAN GTP-binding nuclear protein (Ran).** (A) unique spectrum number, (B) unique peptide number, (C) Score delta (D), Charge, (E) fragment ion mass/Da, (F), peptide mass delta/Da.
(TIF)

Figure S9 **Details of LC-MS analysis of Apolipoprotein-A1.** (A) unique spectrum number, (B) unique peptide number, (C) Score delta (D), Charge, (E) fragment ion mass/Da, (F), peptide mass delta/Da.
(TIF)

Table S1 Inclusion criteria for selection of infertile women.
(DOCX)

Table S2 Exclusion criteria during selection of infertile women subjects.
(DOC)

Table S3 Details of infertile and fertile women.
(TIF)

Table S4 Spot volume of differentially expressed proteins.
(XLS)

Table S5 Details of LC-MS analysis of 9 differentially expressed protein spots.
(XLS)
