# Supplementary figures and images for: Alteration in Endometrial Proteins during Early- and Mid-Secretory Phases of the Cycle in Women with Unexplained Infertility
Source: PLoS One. 2014 Nov 18;9(11):e111687. doi: 10.1371/journal.pone.0111687 (PMC4236019; doi:10.1371/journal.pone.0111687)

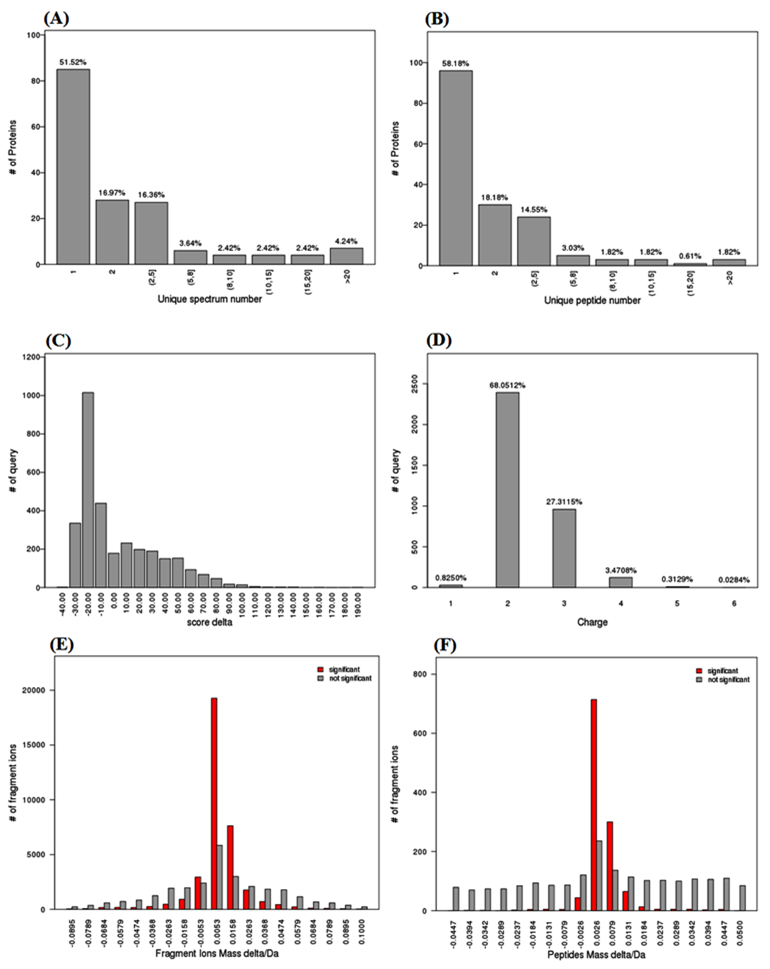

Supplement: Figure S1 — Details of LC-MS analysis of Tubulin-polymerization promoting protein family member-3. (A) unique spectrum number, (B) unique peptide number, (C) Score delta (D), Charge, (E) fragment ion mass/Da, (F), peptide mass delta/Da. (TIF) [file pone.0111687.s001.tif]

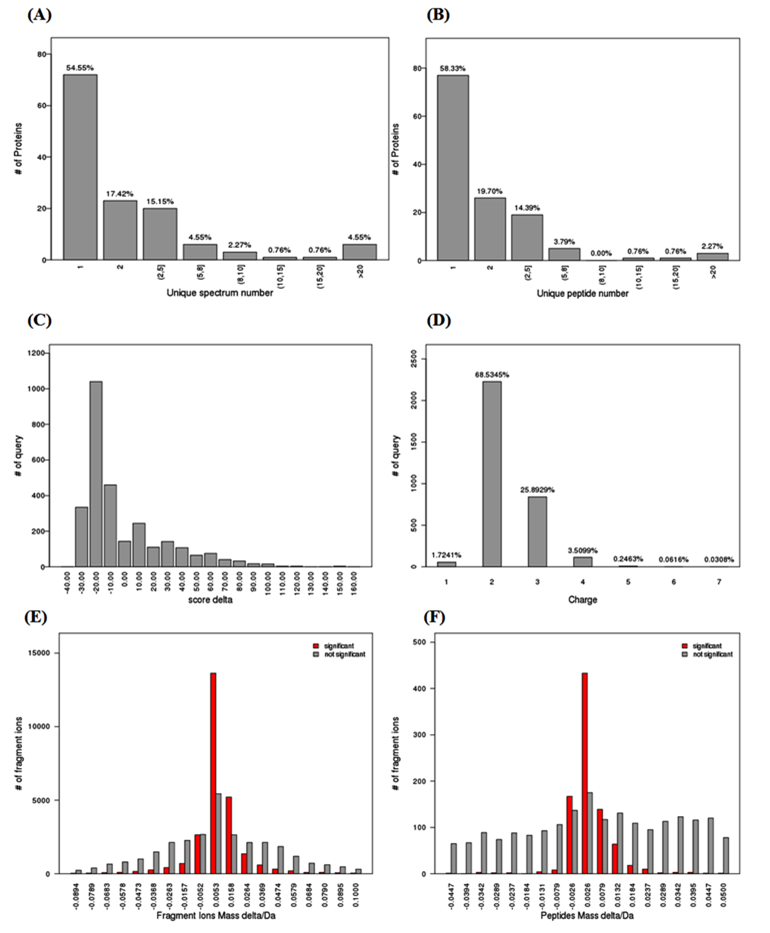

Supplement: Figure S2 — Details of LC-MS analysis of Ras-related protein Rap-1b. (A) unique spectrum number, (B) unique peptide number, (C) Score delta (D), Charge, (E) fragment ion mass/Da, (F), peptide mass delta/Da. (TIF) [file pone.0111687.s002.tif]

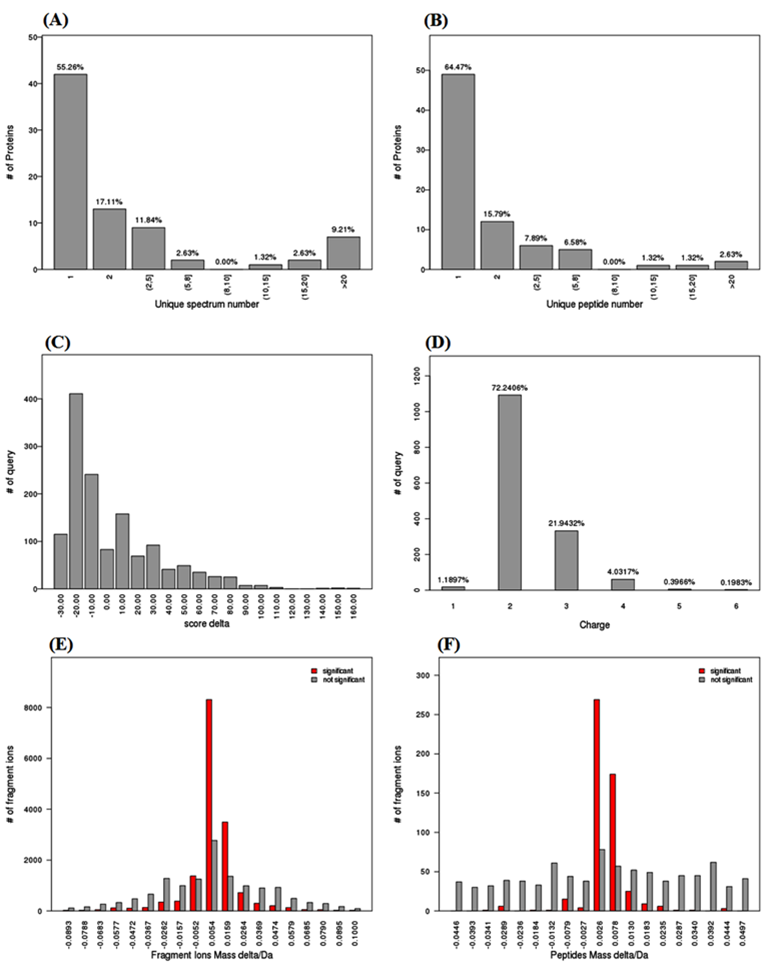

Supplement: Figure S3 — Details of LC-MS analysis of Superoxide dismutase [Cu-Zn]. (A) unique spectrum number, (B) unique peptide number, (C) Score delta (D), Charge, (E) fragment ion mass/Da, (F), peptide mass delta/Da. (TIF) [file pone.0111687.s003.tif]

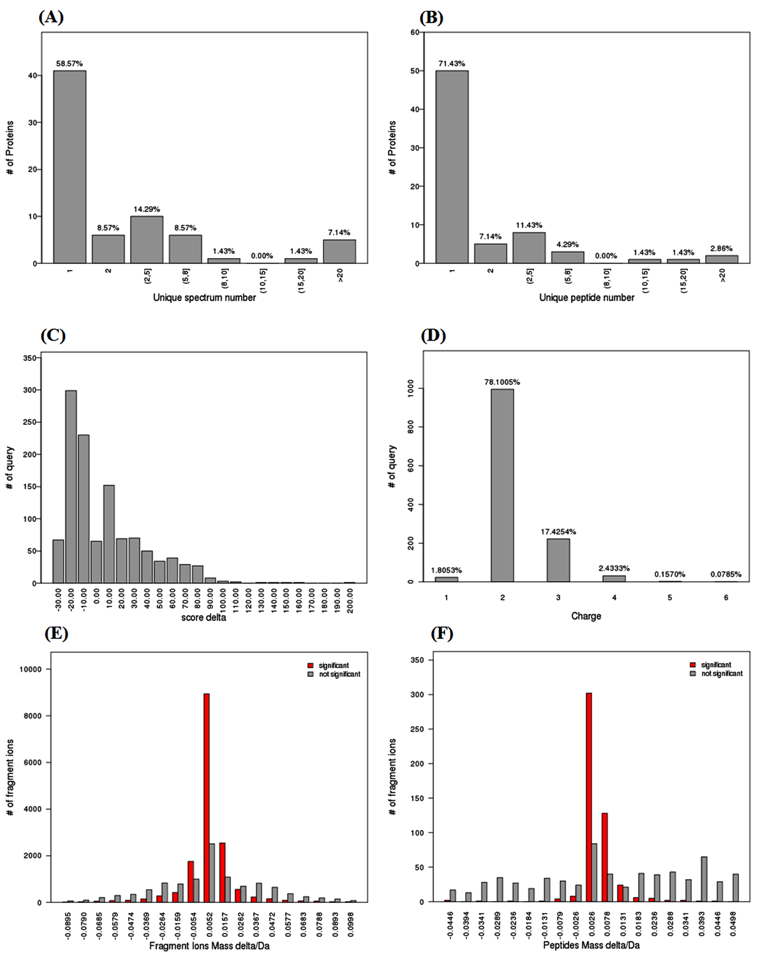

Supplement: Figure S4 — Details of LC-MS analysis of Sorcin. (A) unique spectrum number, (B) unique peptide number, (C) Score delta (D), Charge, (E) fragment ion mass/Da, (F), peptide mass delta/Da. (TIF) [file pone.0111687.s004.tif]

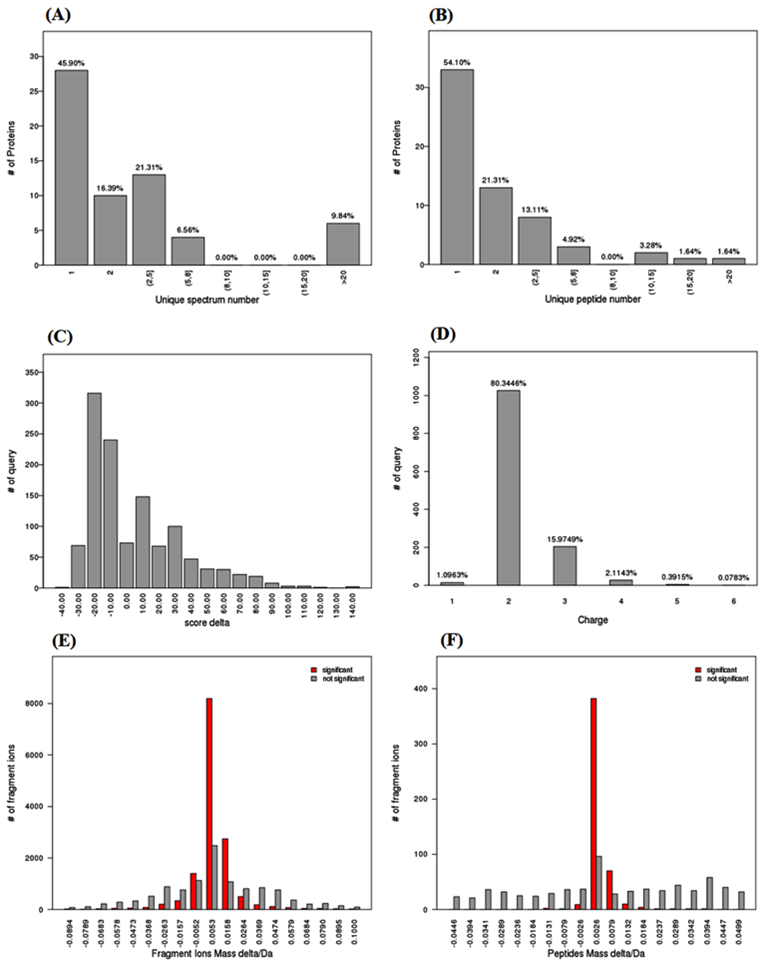

Supplement: Figure S5 — Details of LC-MS analysis of Cofilin-1. (A) unique spectrum number, (B) unique peptide number, (C) Score delta (D), Charge, (E) fragment ion mass/Da, (F), peptide mass delta/Da. (TIF) [file pone.0111687.s005.tif]

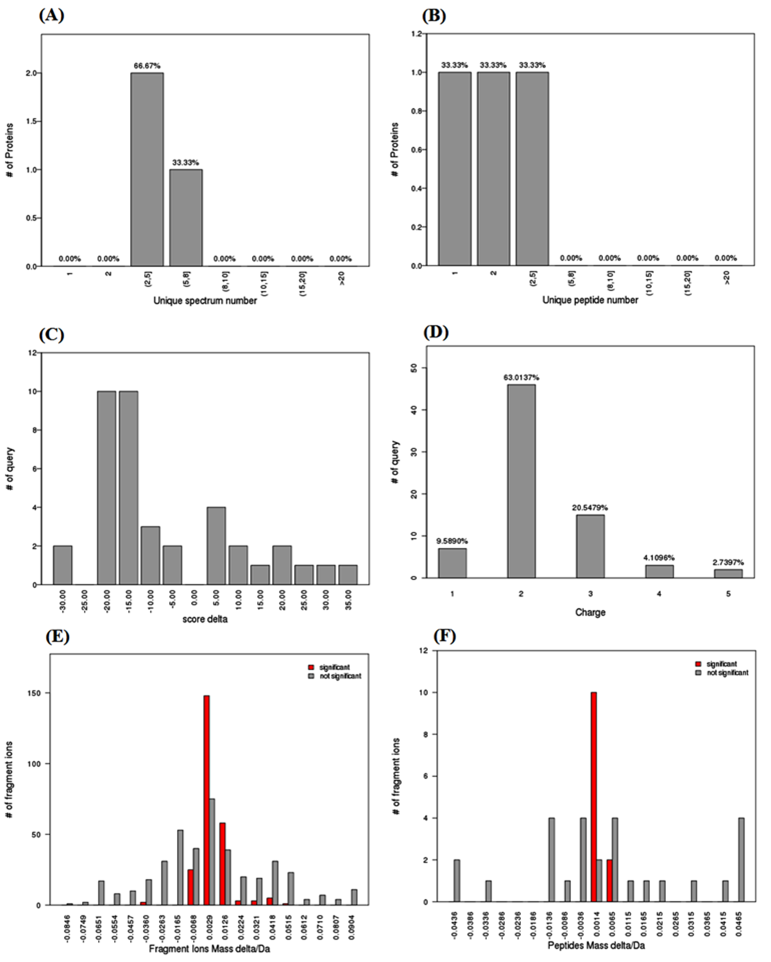

Supplement: Figure S6 — Details of LC-MS analysis of Proteasome subunit alpha type-5. (A) unique spectrum number, (B) unique peptide number, (C) Score delta (D), Charge, (E) fragment ion mass/Da, (F), peptide mass delta/Da. (TIF) [file pone.0111687.s006.tif]

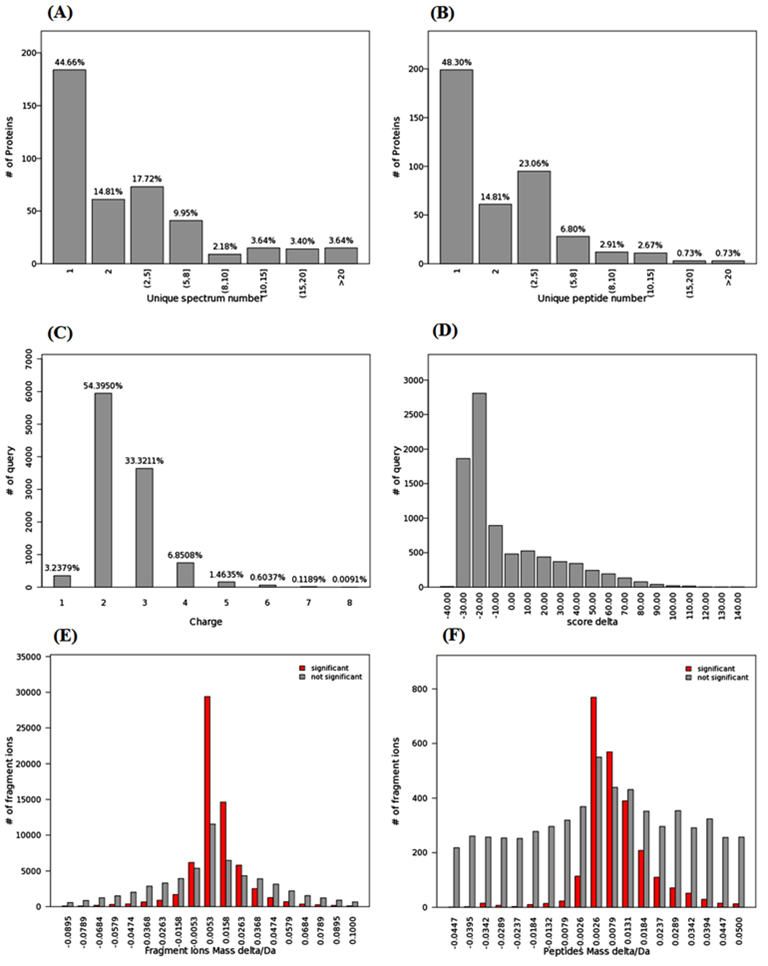

Supplement: Figure S7 — Details of LC-MS analysis of Protein disulfide isomerase A3. (A) unique spectrum number, (B) unique peptide number, (C) Score delta (D), Charge, (E) fragment ion mass/Da, (F), peptide mass delta/Da. (TIF) [file pone.0111687.s007.tif]

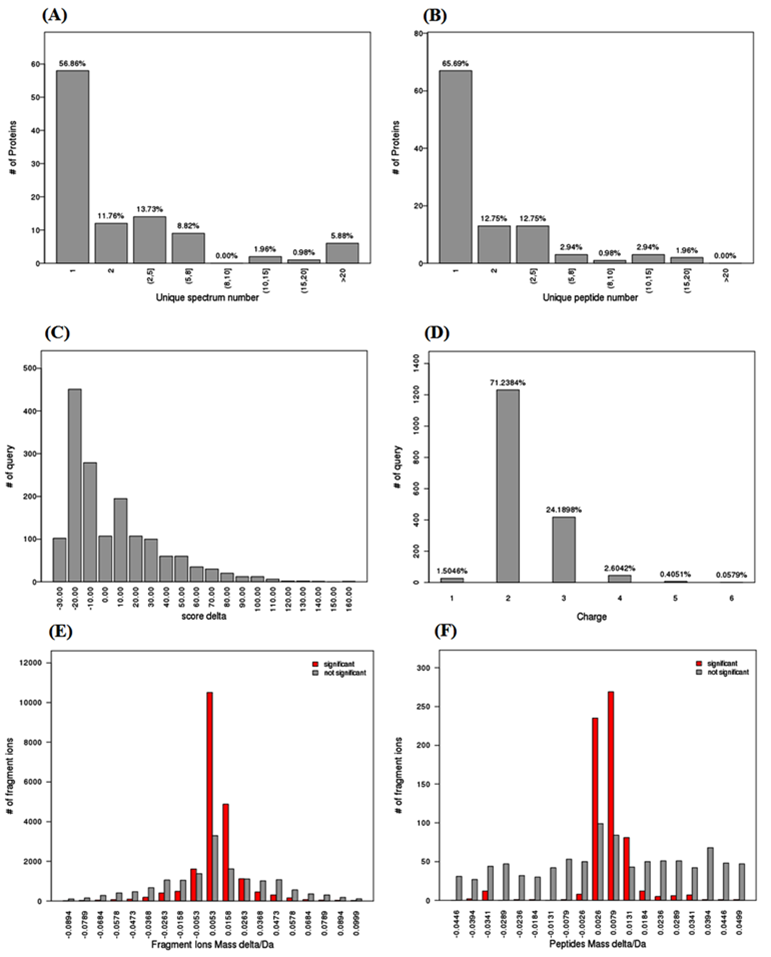

Supplement: Figure S8 — Details of LC-MS analysis of RAN GTP-binding nuclear protein (Ran). (A) unique spectrum number, (B) unique peptide number, (C) Score delta (D), Charge, (E) fragment ion mass/Da, (F), peptide mass delta/Da. (TIF) [file pone.0111687.s008.tif]

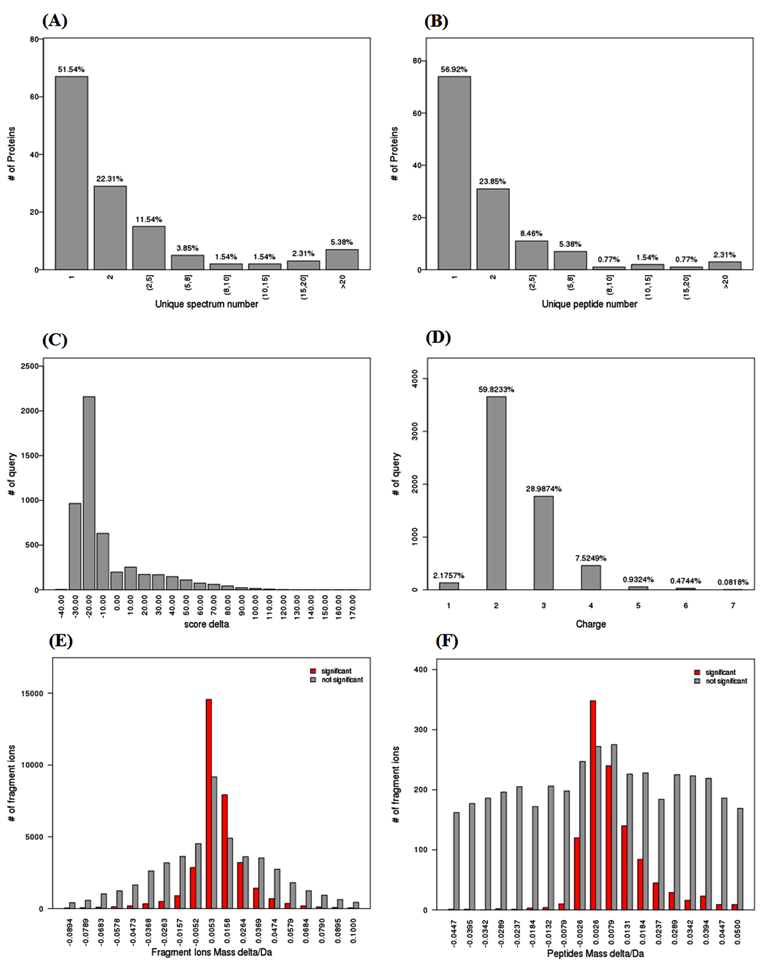

Supplement: Figure S9 — Details of LC-MS analysis of Apolipoprotein-A1. (A) unique spectrum number, (B) unique peptide number, (C) Score delta (D), Charge, (E) fragment ion mass/Da, (F), peptide mass delta/Da. (TIF) [file pone.0111687.s009.tif]

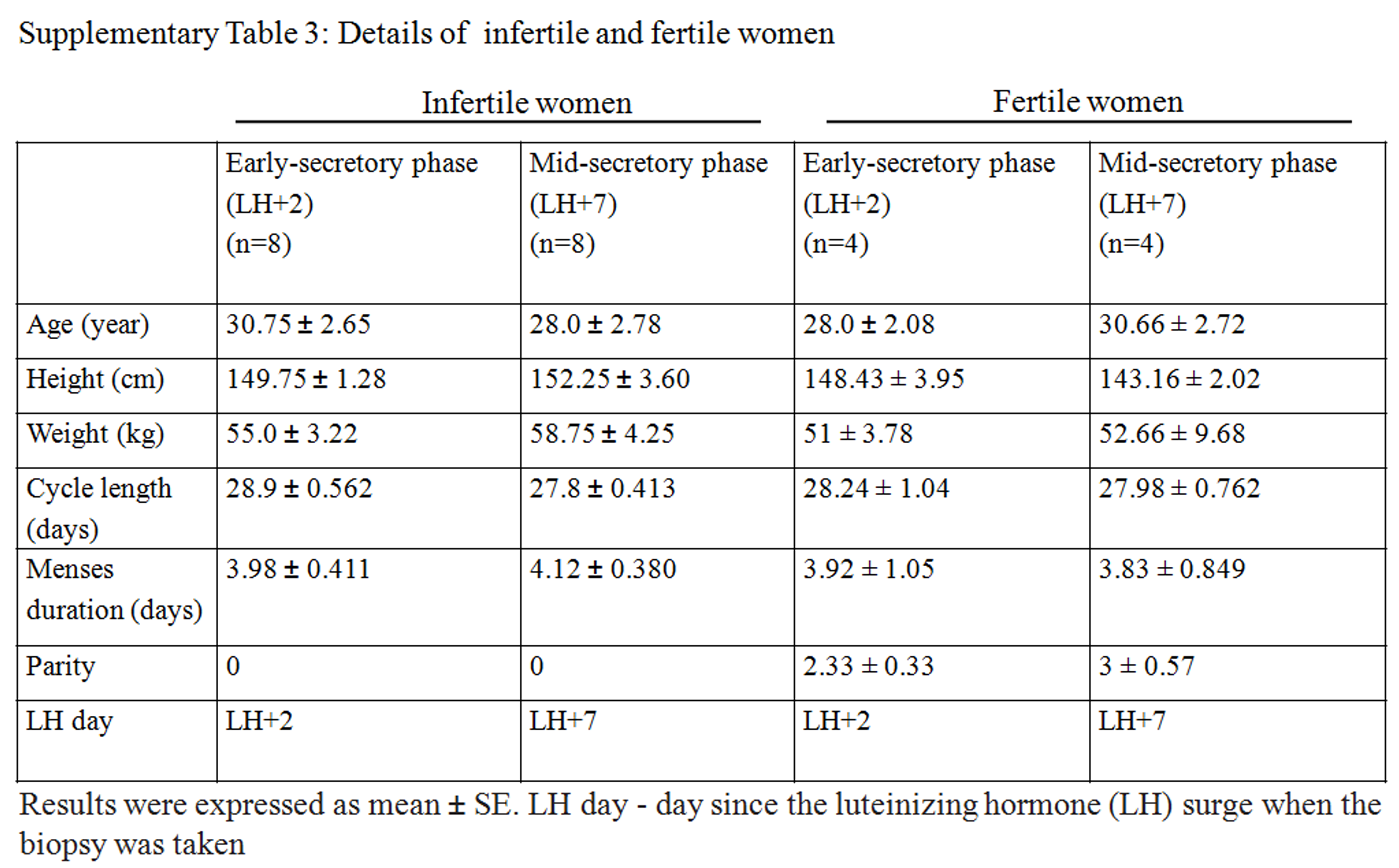

Supplement: Table S3 — Details of infertile and fertile women. (TIF) [file pone.0111687.s012.tif]
