# Supplementary material for: Alteration in Endometrial Proteins during Early- and Mid-Secretory Phases of the Cycle in Women with Unexplained Infertility
Source: PLoS One. 2014 Nov 18;9(11):e111687. doi: 10.1371/journal.pone.0111687 (PMC4236019; doi:10.1371/journal.pone.0111687)
Supplement: Table S1 — Inclusion criteria for selection of infertile women. (DOCX) [file pone.0111687.s010.docx]

Supplementary Table 1: Inclusion criteria for selection of infertile women

| **S. No.** | **Parameter investigated*** | **Status** |
| --- | --- | --- |
| 1 | Cycle length | normal |
| 2 | Fallopian tube (HSG/SSG, laproscopy and stereoscopy) | normal and functioning |
| 3 | Ovulatory function | normal |
| 4 | Bacterial vaginosis | absent |
| 5 | Test for diabetes | negative |
| 6 | Test for tuberculosis | negative |
| 7 | Male partner sperm count | normal |

*All 24 infertile subjects were evaluated for these parameters. All 24 subjects used in the study had not conceived for atleast one year.
