# Supplementary material for: Alteration in Endometrial Proteins during Early- and Mid-Secretory Phases of the Cycle in Women with Unexplained Infertility
Source: PLoS One. 2014 Nov 18;9(11):e111687. doi: 10.1371/journal.pone.0111687 (PMC4236019; doi:10.1371/journal.pone.0111687)
Supplement: Table S2 — Exclusion criteria during selection of infertile women subjects. (DOC) [file pone.0111687.s011.doc]

Supplementary Table 2: Exclusion criteria during selection of infertile women subjects

| **S. No.** | **Exclusion criteria**** |
| --- | --- |
| 1 | Leiomyomas |
| 2 | Polycystic ovarian syndrome |
| 3 | Endometriosis |
| 4 | Acute infection PID |
| 5 | Vaginitis |
| 6 | Male factor infertility |
| 7 | Steroid hormone therapy in the last six months |

** Subjects with these anomalies were omitted from the study
